# Selfie-Related Incidents: Narrative Review and Media Content Analysis

**DOI:** 10.2196/47202

**Published:** 2023-09-27

**Authors:** Samuel Cornell, Robert Brander, Amy Peden

**Affiliations:** 1 School of Population Health University of New South Wales Sydney Australia; 2 Beach Safety Research Group University of New South Wales Sydney Australia; 3 School of Environmental, Earth, and Biological Sciences University of New South Wales Sydney Australia

**Keywords:** selfie, aquatic locations, death, injury, risk, communication, social media, drowning, mobile phone

## Abstract

**Background:**

Selfie-related injury has become a public health concern amid the near ubiquitous use of smartphones and social media apps. Of particular concern are selfie-related deaths at aquatic locations; areas often frequented because of their photogenic allure. Unfortunately, such places exhibit hazards inherent with their environment.

**Objective:**

This study aimed to ascertain current evidence regarding selfie-related injuries and recommended risk treatment measures in the academic literature as well as how selfie-related injuries and deaths were being reported by the media, allowing us to identify key challenges facing land managers and public health practitioners in mitigating selfie-related injuries and deaths.

**Methods:**

Between October and December 2022, we performed a narrative review of peer-reviewed literature published since January 2011. Literature was screened to identify causal factors implicated in selfie-related deaths and injuries, as well as risk treatments recommended. Furthermore, we used an environmental scan methodology to search for media reports of selfie-related injuries and deaths at aquatic locations in Australia and the United States. Individual cases of selfie-related aquatic injuries and deaths sourced from news reports were analyzed to assess epidemiological characteristics, and a thematic content analysis was conducted to identify key themes of news reporting on selfie-related deaths and injuries.

**Results:**

In total, 5 peer-reviewed studies were included. Four studies identified falls from height as the most common injury mechanism in selfie incidents. Drowning was the second most common cause of death. Recommended risk treatments were limited but included the adoption of “no selfie zones,” physical barriers, signage, and provision of information on dangerous locations to social media users. In total, 12 cases were identified from media reports (4 injuries and 8 fatalities; 7 in Australia and 5 in the United States). The mean age of the reported victims was 22.1 (SD 6.93) years with victims more likely to be female tourists. Content analysis revealed 3 key themes from media reports: “blame,” “warning,” and “prevention and education.” Few media reports (n=8) provided safety recommendations.

**Conclusions:**

The selfie-related incident phenomenon should be viewed as a public health problem that requires a public health risk communication response. To date, little attention has been paid to averting selfie-related incidents through behavior change methodologies or direct messaging to users, including through social media apps. Although previous research has recommended “no selfie zones,” barriers, and signage as ways to prevent selfie incidents, our results suggest this may not be enough, and it may be prudent to also engage in direct safety messaging to social media users. Media reporting of selfie incidents should focus on preventive messaging rather than blame or warning.

## Introduction

### Background

Social media use has grown exponentially since the development of the first mobile apps, such as Instagram and Facebook, and the widespread availability and affordability of smartphones [1]. According to the Pew Research Center [2], in 2015, 65% of American adults used at least one social media platform—a 10-fold increase from 2005. By 2022, that figure had increased to 74% of all US adults [3]. Similar rates of social media use have been reported in Australia [4].

The concurrent growth of social media and smartphone use, and the increased amount of time that people spend using web-based and internet-connected platforms, has given rise to myriad new sociotechnological trends. For most people, every aspect of human life is lived in stark contrast to presocial media and presmartphone use. These domains include travel and tourism [5], food consumption [6], political involvement and affiliation [7], health and fitness [8], ideological and religious following [9], conspiratorial thinking [10], group identification [11], and, pivotally, communication [12-15].

One significant contemporary issue stemming from these rapid technological changes to communication and connectivity has been the phenomenon of taking photographs for the purpose of sharing on social media, and, in particular, the taking of selfies [16-18], which are photographs taken by the photographer either alone or with others. Selfie taking has been discussed in psychological and philosophical literature to ascertain and explain the underlying reasons for the action itself, with explanations ranging from narcissism [19], to selfies being a modern art form and simply a new way of personal expression when communicating [20].

Selfie taking is known to be a cause of injuries and fatalities in many countries around the world due to the distraction of the selfie taker. This may be considered one aspect of maladaptive, or problematic, mobile phone use [21]. Maladaptive or problematic phone use can lead to symptoms of distress or separation anxiety when the phone user is unable to use their device [22,23]. Furthermore, maladaptive phone use can lead to adverse consequences for the phone user as they go about their daily life, including when driving [24] or walking [25]. Phone use while driving can cause dangerous levels of distraction and cause road traffic accidents leading to injuries or deaths. In Australia, 61% of drivers have reported using their phones while driving. Due to the seriousness of this type of phone use, many jurisdictions have imposed a ban on the use of mobile devices while the user is driving [26].

While the large majority of selfies taken globally are innocuous, and taking a selfie in itself is not an inherently dangerous activity, selfie takers may seek out potentially dangerous locations, involving risky activities or behaviors, in order to capture a more visually enticing selfie [27]. To understand why a person may go to extreme lengths to capture a selfie necessitates understanding the mechanisms of social media and the inbuilt reward systems that users of social media apps partake in. It is imperative to acknowledge a key tenet of social media—that the person using the social media platform is viewed by the social media company as the product. That is, the social media platform, which is free for a person to use at their discretion, is a web-based system that provides advertising companies with the ability to market to users (the product) of the platform in a personalized manner. This surmises the “attention economy” [28] and highlights the motive for a social media platform to keep its users as active and engaged as possible.

Social media platforms keep users engaged with key metrics including follower counts, “likes” or “loves,” and comments from other users, along with notifications to direct users back to the app when there is a change to one of these metrics. Proprietary algorithms serve to dictate what type of content becomes “viral” (ie, spreads widely on the platform for a period of time). It is understood that certain types of photographic content are shared more widely than others and that by mixing the key ingredients of viral photography effectively, a user can share a photo that increases these metrics [29]. In turn, due to the monetization of social media and the rise of “influencers” (users who gain a large following, advertise products on behalf of companies, and become widely known), many users are compelled to create photo content that is seen by the maximum number of users [30,31].

Thus, users may take selfies with the aim of distributing them on social media. Users have discovered that selfies that picture the sublime, risky activities, poses, and beautiful scenes have the propensity to deliver them the elusive “instafame,” which they seek [32]. Selfies taken in aquatic locations, often picturing waterfalls, and cliff edges, are particularly enticing to selfie takers as these photographs contain all the essential ingredients for viral content [33].

Aquatic locations are important to understand as sites of the selfie problem due to their wide-ranging photographic appeal across ages. Furthermore, aquatic locations, being picturesque and with varied topography, are often in remote areas with fewer services available to perform a rescue. Aquatic locations present a wide array of geographic hazards, from cliffs and waterfalls to dangerous currents, waves, drop-offs, and slippery surfaces. They are often difficult, dangerous, and costly for emergency services to reach when performing rescues or retrievals [34-37].

Selfie-related injuries and deaths have been reported in the media, most commonly in India, the United States, and Russia [38]. The mean age of victims of selfie-related injury has been reported as 23-24 years [39,40], and most victims are male with reports of up to 72.5% of selfie-related injuries and deaths involving men or boys [41]. Across the globe, aquatic locations are prevalent geographic hotspots for selfie-related injuries and deaths. At these aquatic locations, falling from height and drowning are the most common mechanisms of injury and death and often occur together [42].

Research has found that male selfie takers are more likely to be involved in selfie-related deaths and injuries even though female selfie takers take more selfies on average [43]. The underlying reason for this disparity in selfie taking in relationship to casualty rates may be that male selfie takers take riskier selfies, such as at cliffs and aquatic locations and therefore are at greater risk of injury or death [38]. Men and boys are understood to engage in riskier behaviors more generally, and this risk-taking seems to translate into risky selfies [44-46].

### Aims

While we acknowledge that selfies are simply one aspect of contemporary photography and social media sharing, this study examines the phenomenon of selfie taking as a cause of injury and death. This study amalgamates data from both published academic literature and news reports to provide a comprehensive overview of the selfie problem as it affects Australia and the United States with an emphasis on aquatic locations. Previous studies investigating selfie-related injuries and deaths have taken data from Wikipedia and Twitter images [47,48], which may underestimate the true number of aquatic selfie injuries and deaths that have occurred in Australia and the United States. Our study incorporates records from news reports; thus, we were able to get a wider range of information related to selfie deaths. The study aimed to ascertain how selfie-related injuries and deaths were being reported by way of a media-based content analysis. This type of qualitative process, alongside a review of the current academic literature on this topic, will enable us to highlight the key challenges that face land managers and public health practitioners to mitigate the incidence of selfie-related injuries and deaths.

## Methods

### Study Design

We performed a literature review for academic papers that had been published in the field [49] and used an environmental scan methodology [50] adapted from previous studies [51,52]. The environmental scan involves systematically searching publicly accessible websites, including news reports, to capture resources that may not be included in academic journal papers. We further conducted a review of the academic literature using the databases Scopus, Web of Science, and PubMed, as well as Google Scholar. We conducted a content analysis on all media papers that were included in the study [53]. No participants were recruited for this study, and data were collected from publicly available websites. As such, ethics approval was not required. The study was conducted between October 31 and December 21, 2022, using only publicly available media reports and academic papers available on the Scopus database, Web of Science, PubMed, or through Google Scholar.

### Literature Review Search Strategy

For the academic literature, 1 author (SC) searched the Scopus database, Web of Science, PubMed, and Google Scholar. The search terms included Selfie* with Death*, Fall*, Injury*, *Mortality*, *Risky*, *Deadly*, Drown*, Accident*, and *Accidents*[mesh]. Papers were read first by title and abstract. If the abstract provided details on the epidemiological incidence or prevalence on selfie-related injuries or deaths, then the paper was read in full by SC and included in the review if it contained reference to any of the aquatic search terms and selfie-related deaths or injuries in these aquatic areas.

### Literature Review Inclusion and Exclusion Criteria

The inclusion and exclusion criteria are included in Textbox 1.

**Inclusion and exclusion criteria for peer-reviewed literature.**

**Inclusion criteria**

- Paper type: Peer-reviewed original research and review papers
- Language: English
- Database: Available on Google Scholar Scopus, Web of Science, or PubMed
- Timeframe: Published since January 2011
- Content: Reported selfie-related injuries or deaths in aquatic environments
- Location: Reported on incidents in Australia and the United States

**Exclusion criteria**

- Paper type: Conference abstracts, grey literature, reports
- Language: Papers not in English
- Timeframe: Published before January 2011
- Content: Did not report on selfie-related incidents in aquatic environments
- Location: Did not report on incidents in Australia and the United States

### Environmental Scan Search Strategy

We used two main strategies to identify news articles containing eligible reports: a search using Google and a manual search of a Wikipedia page repository, which is frequently updated with selfie-related injuries and death news reports [54].

In total, 7 search terms were used to capture information about selfie-related aquatic deaths and injuries (*lake*, *beach*, *cliff*, *coast*, *drowning*, *waterfall*, and *river*). Each of these terms was combined with the term *selfie* and *injury* OR *death* (eg, *selfie death* AND *waterfall*), resulting in 14 unique searches. The word selfie is so ubiquitous on the web and society at large (having been the Oxford Dictionary’s “word of the year” in 2013) that it was deemed unnecessary to include *self-photography* as a term, as this is rarely used, if ever, by media outlets or social media users. The first 50 results from the first page of each Google search were considered, as previous research has indicated minimal new relevant results after this [55].

One author (SC) manually searched the Wikipedia page “List of Selfie Related Deaths and Injuries.” Media papers that referred to aquatic deaths were included and checked for duplication with results obtained from Google searches for each term. Two authors (SC and RB) then conducted searches using Google. Each reviewer used the web browser Google Chrome (Alphabet Inc) with an incognito browser window and reset the cache in their browser windows before each search to minimize the effect of Google search optimization. The first 50 results from the first page of each Google search were exported to Microsoft Excel (Microsoft Corp) using the SEOQuake [56] browser extension, with some searches yielding fewer than 50 results on the first page. All search terms yielded the same number of results for both reviewers. Results from both search strategies (known Wikipedia repository and Google search) were combined, and duplicate results were removed. All results were screened independently for inclusion by 2 authors. There were no discrepancies.

News articles that met the inclusion criteria were not excluded, were downloaded using the web browser extension, NCapture (QSR International), and were then imported into NVivo (QSR International). Exact duplicates of news articles were removed; however, news articles that were similar and reporting on the same incident, but not exact replications, were retained.

### Environmental Scan Inclusion and Exclusion Criteria

Inclusion criteria included media reports available on the web and published on any date in English language. News reports must have been freely and publicly available on the web (ie, not behind a paywall). News reports must have been reporting on specific cases of either individual or group selfie-related injuries and deaths in aquatic locations (including cliffs, waterfalls, beaches, rivers, lakes, ponds, and other bodies of water) in Australia or the United States. The injuries or deaths must have been attributed to taking a selfie, either before or during the injury or death. The victims may have been of any nationality, age, or sex and may have spoken any language. Google Search was chosen in lieu of Google News for this media search due to the ability to specify search criteria and download the URLs with a web extension used in studies with a similar environmental scan methodology [52].

Downloaded Google URLs that were not news reports (eg, academic papers, Facebook posts, and blog posts) were immediately excluded. Exclusion criteria also included papers that could not be accessed without a subscription to the news site. These were excluded due to an inability to obtain their data. Photography-related deaths that were not selfie-related and selfie-related injuries not amounting to death were excluded. Selfie deaths were differentiated from deaths due to mobile phones. For example, if a person died accidentally while distracted using a mobile phone, but was not taking a selfie, this was not included. News reports that were reporting on the overall phenomenon of selfie-related deaths or injuries, but not specific incidents, were not included.

### Content Analysis Method

All news articles were read in full by the first author, SC. Coding categories were developed iteratively [57]. News articles were coded and analyzed for topic, framing of argument, overall slant (including whether the paper took a warning tone, prevention tone, or blame tone), mention of prevention activities or warning signs, and direct quotes or position statements by government agencies. The coding framework can be seen in Multimedia Appendix 1.

## Results

### Literature Results

The literature search returned 59 papers from Scopus, 8 from Web of Science, and 15 from PubMed. Google Scholar did not return any other relevant results. After checking each result against the inclusion and exclusion criteria, 5 published studies were included for assessment (Table 1).

Papers were searched for cases of selfie-related deaths in aquatic locations in the United States and Australia. No specific cases were reported in any of the published literature. Papers were further assessed for a number of selfie-related deaths or injuries reported in the United States, Australia, and worldwide. It was unclear from the included papers how many reported selfie-related deaths or injuries were occurring in aquatic locations. One paper, by Jain and Mavani [38], did report deaths and injuries in aquatic locations but did not attribute any deaths or injuries to this in the United States or Australia.

The literature revealed that drowning and falls are the most common mechanisms of death or injury in selfie-related incidents (Table 1). The mean age of victims included in the literature was 23.5 (range 22.94-24.4) years. Men were more likely to be implicated in a selfie-related incident and were more likely to be injured or killed.

**Table 1:** Academic literature search results.

Paper	Authors (year)	Timespan of paper data	Deaths or injuries reported (worldwide), n	Selfie-related deaths or injuries , n			Deaths or injuries reported in aquatic locations, n			Age of victims (years), mean (SD)		Most common mechanism of injury or death (all cases, n)		Preventive recommendations
				United States	Australia	Worldwide		Australia and the United States						
Me, myself and my killfie: characterizing and preventing selfie deaths	Lamba et al (2016) [48]	Mar 2014 to Nov 2016	127	8	1	54		Unclear	Not available		Fall from height (25)		Suggested building technologies that help users identify if a specific location is dangerous for taking selfies and provide information on previous harms that have occurred at a given location.	
Selfies: a boon or bane?	Bansal et al (2018) [41]	Oct 2011 to Nov 2017	259	14	1	70		Unclear	22.94		Drowning (27)		Suggested that “no selfie zones” should be declared at locations popular with tourists, such as bodies of water, mountain peaks, and over tall buildings.	
Media-based clinical research on selfie-related injuries and deaths	Dokur et al (2018) [39]	Dec 2013 and Jan 2017	159	10	1	Unclear		Unclear	23.48 (10.1)		Fall from height (25)		No specific recommendations are provided. There is a call to introduce “drastic measures” to reduce selfie-related deaths and injuries.	
A comprehensive study of worldwide selfie-related accidental mortality: a growing problem of the modern society	Jain and Mavani (2017) [38]	2013-2016	75	6	0	30		0	23.3		Falls from heights (32)		Calls for the implementation on “no selfie zones” at high-risk locations. Suggests that a multifactorial approach by government, law, tourism, psychology, telecom, and social media is adopted.	
Selfie-related deaths using web epidemiological intelligence tool (2008-2021): a cross-sectional study	Linares et al (2021) [40]	2008-2021	379	39	15	83		Unclear	24.4 (11.9)		Falls from heights (50)		No prevention recommendations.	

In summary, Linares et al [40] found that 433 people were involved in selfie-related incidents leading to 379 individual deaths across 292 distinct incidents. Of these, 141 (37%) were identified as tourists. The number of deaths increased from 3 in 2013 to 68 in 2019. Yearly incidents (deaths) decreased during the first 2 years of the COVID-19 pandemic. In the first year of the pandemic, 2020, their decreased, with just 37 cases reported in 2020. In the first 6 months of 2021, 31 cases were reported. The mean age of the fatalities was 24.4 (SD 11.9) years. Geographically, the highest numbers of incidents and selfie deaths were reported in India (n=100, 26%), followed by the United States (n=39, 10%) and Russia (n=33, 9%). The study found that falling from a height was the most common mechanism of incidents and deaths (50%), followed by transportation (29%) and drowning (14%). The mean age of selfie-related death for travelers or tourists was higher than the overall mean. People who died from selfie-related injuries were more likely to be tourists in the United States and Australia and more likely to be local residents in Russia and Brazil. The study did not provide any prevention or intervention recommendations.

Research by Dokur et al [39] analyzed 159 selfie victims from 111 events reported via media sources and found the most frequently reported event type was falling from a height. The average age of the victim was 23.36 (SD 10.1) years. It was determined that students (particularly high school and university students) were predominant (n=84, 53%); the numbers of domestic and foreign (international) tourists were 78 (49%) and 15 (9%), respectively.

With regard to communicating the risk of taking selfies in hazardous locations, the literature differed in its recommendations. Dokur et al [39] did not provide any suggestions for how to avert the risk of taking selfies in remote or natural areas. Bansal et al [41] recommended the creation and implementation of “no selfie zones,” particularly in areas with high tourist traffic, bodies of water, mountains, and even tall buildings in cities. Nevertheless, Bansal et al [41] did not provide recommendations on how best to communicate the danger of taking selfies in hazardous locations.

Lamba et al [48] developed an artificial intelligence–assisted program that can identify dangerous locations to take selfies through image and text input. They suggest that using such a technology may alert selfie takers to locations that are hazardous for selfie activity. The researchers paid particular emphasis to locations with water and height, as research has shown these types of locations to be the most hazardous for selfies. It is unclear how Lamba et al [48] would implement the technology they have created into programs commonly used by selfie takers, such as Instagram, and if they did implement it, at what point the program would alert the selfie takers to the danger they face. Linares et al [40] made suggestions for communicating the risk of taking selfies to travelers but did not provide insights for local populations.

Lamba et al [48] advised that travel medicine practitioners should instruct travelers on safe selfie taking. However, this approach may be too specific to reach large groups who are at risk. Lastly, Jain and Mavani [38] recommended “large-scale trials” to avert selfie-related incidents but did not suggest what these trials should encompass. The authors also cited the implementation of “no selfie zones,” such as in Russia, and recommended an interagency approach to averting more selfie disasters.

### Results for Media Reports

**Figure 1:**
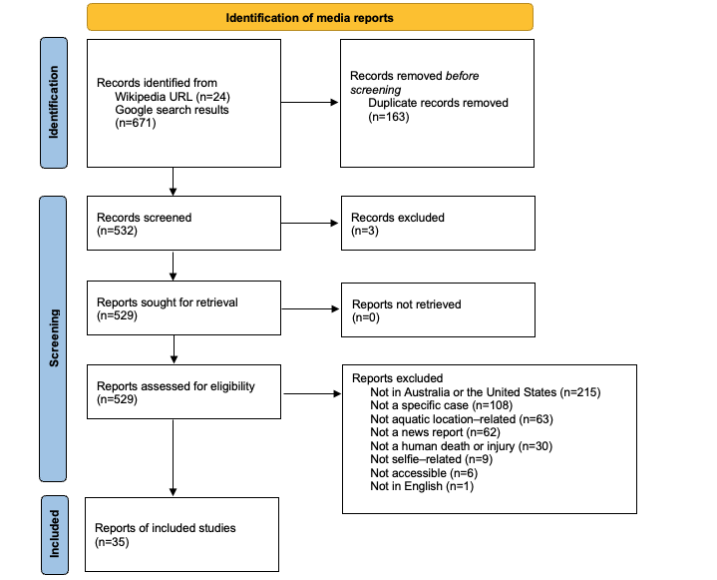
PRISMA (Preferred Reporting Items for Systematic Reviews and Meta-Analyses) diagram outlining identification and inclusion of media search papers. Further details are present in Multimedia Appendix 2.

The Google search resulted in 2 independent pools of 671 URLs, with a total of 510 after removal of duplicates from each search. The Wikipedia search resulted in 24 URLs, 2 of which were removed due to duplication. The combined Google and Wikipedia search resulted in 532 URLs for screening. After applying inclusion and exclusion criteria, 35 URLs were found to meet the criteria for inclusion with 491 exclusions. The reasons for exclusion are detailed in Figure 1. The PRISMA (Preferred Reporting Items for Systematic Reviews and Meta-Analyses) checklist is shown in Multimedia Appendix 2.

### Media Case Reports

Of the 35 included URLs, 12 specific cases were identified (Table 2). This included 7 cases in Australia and 5 in the United States.

Media cases revealed that female selfie takers were more likely to be implicated in a selfie-related incident at an aquatic location in Australia or the United States. The media cases also demonstrated that tourists are an at-risk group of selfie takers. Most media cases reported on selfie incidents at coastal locations (n=7, 58%) and mostly at cliffs (n=8, 66%). At these locations, the selfie incident commonly involved the victim being injured or killed by falling.

**Table 2:** Characteristics of cases identified via media search.

ID	Date (month and year)	Country	Location	Body of water	Land manager responsible	Mechanism of injury or death	Injury or death	Details	Victim	Local or tourist
1	January 2016	Australia	Figure Eight Pools. Royal National Park, New South Wales	Coastal	NSW National Parks	Falls due to wave	Injuries	Multiple injuries including head injuries, cuts, grazes, and blunt trauma	Multiple injuries to approximately 100 people	Tourists
2	March 2017	United States	Bandon, Oregon	Beach or coastal	Local council	Trapped or drowned	Death	Drowned	Female, 14 years	Local
3	May 2018	Australia	The Gap, Albany, Western Australia	Cliffs or coastal	Western Australia National Parks	Fall	Death	Fell down cliff	Male, 20 years	Tourist
4	July 2018	Australia	Cape Solander in Kurnell near Sydney	Cliffs or coastal	NSW National Parks	Fall	Death	Selfie at cliffs leading to fall	Male, 19 years	Tourist
5	September 2018	United States	Lake Superior, Pictured Rocks	Cliffs or lake	US National Park Service	Fall	Death	Fell from rock face into water below while taking a selfie	Female, 32 years	Tourist
6	October 2018	United States	Potomac, Maryland	River	Local council	Fall	Injury	Fell into river	Unclear	Unclear
7	June 2019	United States	Lake Tahoe, California	Lake	US National Park Service	Fall	Death	Fell down waterfall	Unclear	Unclear
8	August 2019	Australia	Diamond Bay Reserve, Sydney	Cliffs or coastal	Local council	Fall	Death	Selfie at cliffs leading to fall	Female, 27 years	Local
9	January 2020	Australia	Diamond Bay Reserve, Sydney	Cliffs or coastal	Local council	Fall	Death	Fell from a 98-foot-high cliff while taking a selfie	Female, 21 years	Tourist
10	April 2020	Australia	Diamond Bay Reserve, Sydney	Cliffs or coastal	Local council	Fall	Injury	Fell down cliff	Female, 16 years	Local
11	July 2021	Australia	Kangaroo Point Cliffs, Brisbane	Cliffs or river	Local council	Fall	Death	Slipped down cliff face while taking a selfie	Female, 33 years	Tourist
12	May 2022	United States	Raven Cliff Falls, Georgia	Cliffs or river	US Forest Service	Fall	Injury	Fell down cliff	Female, 17 years	Unclear

### Content Analysis of Media Reports

#### Overview

Analysis of the media reports revealed three major themes: (1) blame, (2) warning, and (3) prevention and education (Table 3).

**Table 3:** Example content from analysis of the 24 media reports that were assessed for inclusion.

Report number			Example content
**Blame**			
	6	“Said the incident was ‘extremely sad’ and urged those frequenting the popular clifftop viewing area not to endanger themselves by climbing fences or venturing too close to the edge. ‘You’re putting your life at risk and why—for a photo? It’s not that important.’”	
	17	“The council agreed to investigate ways to restrict or deter movement around the area, including the installation of CCTV, more physical barriers, multilingual signage and more frequent ranger patrols—particularly on weekends.” “There is an ongoing and justifiable concern that visitors are irresponsibly endangering themselves and others by crossing over fencing and boundary lines and positioning themselves on the cliff ledge,” the motion noted.	
	21	“A teenage girl skylarking with friends in the dead of the night at a selfie hotspot has miraculously survived a 15-metre drop just months after a British model plunged to her death at the notorious cliff face.”	
**Warning**			
	6	“Visitors warned to take care—This is the second fatal incident at Cape Solander in six weeks, after a man aged in his 30s died in similar circumstances last month.”	
	10	“‘Swimming is prohibited in this area of the Potomac River,’ he said. ‘So we’re constantly warning people to stay away from the water—it’s dangerous.’”	
	12	“‘This is a sad reminder to be cautious when taking selfies and other photos in dangerous areas. Don’t underestimate the power of waterfalls, rivers, and cold-water temperatures,’ the fire district wrote in a Facebook post.”	
**Prevention and education**			
	6	“I really want to find out exactly where...it happened, was there fencing? Was there any signage there? And if we’ve got to look as a community and as a council and a government to spell it out more, we will spell it out more. We will probably need to educate people more.”	
	18	“Signs warn tourists to stay away from the cliff edge and the height of fences in the area had been increased, according to a local mayor.”	
	20	“There are reportedly signs in the area warning people to stay away from the edge of the cliff. Waverley Mayor Paula Masselos told the BBC that the city has already increased the height of fences in the area and added additional warning signs in other languages.”	

#### Theme 1: Blame

In total, 8 (33%) papers took a stance of victim blaming (such as placing fault on the victims) when reporting the selfie-related injury or death. Blame was often placed upon the victim’s actions and referred to the situation that the victim had placed themselves in. Papers reported on the victim’s activities prior to taking the selfie and often reported on the victim’s lifestyle, hobbies, or other pursuits. Papers reported whether the victim had been with friends or was solo prior to taking the selfie. Papers referred to drugs, alcohol, and other risky behaviors in relation to the selfie incident and placed an emphasis on personal responsibility and safety.

#### Theme 2: Warning

In total, 8 (33%) papers took a warning tone in which the primary focus of the paper aimed to convey the inherent danger of risky behaviors, such as taking selfies, around aquatic locations. Papers that took a warning tone often referred to the geographical hazards present in these locations. Such papers also referred to signs or barriers that are present in these areas and would segue to blame if such signs or barriers had been dismissed or circumnavigated.

#### Theme 3: Prevention and Education

In total, 8 (33%) papers took a prevention and education stance and provided more information on the local authorities’ viewpoint on the matter of providing information to the public about the dangers of aquatic locations for taking selfies. Nevertheless, analysis of the papers revealed a dearth of preventive information being conveyed. Education on how to stay safe around cliff edges and other aquatic areas while taking photographs was generally absent. The papers did not provide any supportive information on how to stay safe at these locations when taking selfies.

## Discussion

### Principal Findings

Selfies, a phenomenon of the modern age and largely driven by social media use, are a public health concern that is not going away. As an emerging issue, there is currently a scarcity of peer-reviewed literature on the topic. To address this, we examined both peer-reviewed literature and media reports of selfie-related injuries and deaths across Australia and the United States to inform preventive efforts.

### What Do We Currently Know About Selfie-Related Injuries and Deaths in Aquatic Areas?

We identified differences in selfie-related deaths in aquatic locations in Australia and the United States when compared to other countries. In Australia and the United States, selfie-related deaths in aquatic locations most commonly involve falls from cliff edges in coastal settings, with drowning a very prevalent contributory mechanism of death, but not the primary cause in most of the literature [38,39,41,47,58,59]. This is in contrast with other locations, such as India, where the most common location for selfie-related aquatic deaths and injuries is bodies of water including rivers and lakes, and the mechanism of death is drowning [38]. In other locations with high numbers of selfie-related deaths, such as Russia, the most common locations are not aquatic or “natural” but rather man-made structures from which people fall when performing “daredevil” stunts [42]. Furthermore, in countries including India and Pakistan, groups of selfie takers more commonly drown together, whereas in countries like Australia and the United States, selfie takers tend to be involved in incidents while alone.

We found that both the academic literature and the media generally fail to provide prevention recommendations or proposals for strategies to alleviate the incidence of selfie-related deaths and injuries. Some previous research, such as by Lamba et al [47], called for the development of technological solutions to the problem but did not specify any particular mechanisms.

Although previous research, such as Bansal et al [41] and Jain and Mavani [38], has recommended “no selfie zones,” barriers, and signage as ways to prevent selfie incidents, little attention has been paid to averting selfie-related incidents through behavior change methodologies or direct messaging to users, such as through social media apps. Our results suggest it may be prudent to engage in direct safety messaging to social media users that would communicate risks in a way social media users are more familiar with. Few strategies have been recommended to attenuate the rise of selfie-related deaths and injuries, and crucially there has not been any evaluation of strategies to mitigate the problem. This has left land-managing organizations trying to find solutions to the selfie issue with no evidence to support their projects.

The academic literature that was included in this study did not provide detailed information on individual incidents, making it difficult to ascertain the specific circumstances. This necessitated the consideration of news reports of relevant incidents in Australia and the United States. The media cases allowed us to determine the demographic characteristics of the people involved in the incidents that could then be reported distinctly.

Our study of media cases found that young women and girls were more likely to be implicated in selfie-related injuries and deaths at aquatic locations in Australia and the United States. This finding contrasts with other research into selfie incidents, which showed that, globally, young men and boys are more likely to be involved in a selfie-related incident, even though, on average, women and girls take more selfies than men and boys [43]. Our finding may contrast with other research due to the particular interest we have taken both geographically (Australia and the United States) and in terms of the type of incident (aquatic); furthermore, our sample size was small—but only due to the specific nature of our query. The literature reviewed in this study shows that the mean age of a victim in a selfie-related death was 23.5 years; however, our analysis of the media reports revealed a mean age of 22.1 (SD 6.61) years. This may be due to a higher proportion of tourists identified in the media cases for these locations, whereas the cases reported in the literature and media for selfie incidents in places such as India, Russia, and Pakistan more often identify locals. This suggests that future research in Australia and the United States should evaluate selfie use among travelers and potentially take a closer look at why women and girls are implicated in aquatic-related selfie deaths in these countries more than men and boys.

We performed a qualitative content analysis of included media reports. Media reports did not always provide comments from emergency services or land managers but were likely to report comments from the victim’s family or friends from an emotional perspective. This reporting did little to convey information on prevention or education but was more likely to sensationalize and prioritize emotional impact. Blame and warning tones were conveyed through the reporters’ own views. When prevention and education messages were present, they were more likely to be comments from emergency services and land managers. This finding is in line with research conducted on news reporting epidemics, which found that emotion-laden news increased *perceptions of severity* of the issue but did not increase *fear* or *personal vulnerability* [60]. News that reports with high levels of emotion may not be the most appropriate to inform and educate the public on the objective risks of taking selfies in hazardous locations.

Victim blaming, as illustrated within the news reports assessed in this research, is an ideological concept that involves the “victim” or casualty of an act, such as an injury, or crime, being held either entirely or partially at fault and therefore to “blame.” Victim blaming is a psychological phenomenon that is highly prevalent in human society and can lead to those affected by an insult, injury, death, or crime to be blamed for the actions that occurred, regardless of their own culpability or capacity to foresee or avert the actions they were involved in [61,62]. Victim blaming enables the perpetrators or the institutional factors implicit in the victim’s demise to be taken for granted and therefore maintain the status quo. In this sense, victims of selfie taking behavior are seen as getting “what they deserve” (the just-world hypothesis [63]): their injuries, or even deaths, are justified [64].

### What Can Be Done About Reducing Selfie-Related Injury and Deaths?

Our study has highlighted a case to be made for seeing the selfie phenomenon through a public health lens. Taking a public health injury prevention approach to the phenomenon of selfie-related deaths and injuries means treating it as an issue that has been seen as a social trend or norm but that can be harmful and thus requires effective risk communication, education, and harm reduction practices. This approach naturally involves a public health policy approach to facilitate the implementation of effective countermeasures to the issue at hand; crucially, it involves the translation of research evidence of the harm into impactful implementation by public health prevention practitioners [65].

Currently, there is little literature on how to respond to dangerous selfie taking using risk communication methods. Furthermore, media reports on selfie incidents often disregard prevention, education, and communication and favor reporting, which seeks to blame or warn. We recommend media outlets should aim to convey a prevention-focused message in their reporting to educate and inform rather than warn or blame. Researchers should devise co-designed messages with social media end users who take selfies to share media with generic safety and prevention messages that resonate with, and inform, the target audience.

Countries such as Australia and the United States that see large numbers of tourists each year who frequent the countries’ national parks and other scenic, often remote, areas need to devise strategies to effectively communicate the danger of taking selfies, especially when alone, in natural areas. It may be necessary for land management organizations such as national parks and local councils to devise specific communication and engineering strategies that are tailored to their locales to prevent selfie-related injuries and deaths. There is also a need for researchers to engage with land managers to better evaluate the problem and acquire location-specific incident data that are difficult to attain.

A high proportion of selfie-related deaths in Australia are from travelers [40], whereas in the United States, there is a more even distribution between locals and travelers. Messaging needs to be tailored to the jurisdiction that is being considered. It is therefore imperative to understand the key demographics involved in selfie-related incidents at a given location. In addition to this, it is necessary to evaluate effective risk communication strategies that best resonate with selfie takers depending on their location and demographic. Signs and barriers may simply not be enough. Land managers, alongside public health practitioners and injury prevention researchers, must devise strategies to “meet the people where they are” [66], which, in the case of the selfie phenomenon, is often social media.

### What Knowledge Gaps Remain Around the Burden and Prevention of Selfie-Related Injuries and Deaths?

There has been no published research that evaluates any kind of intervention strategy to alleviate the incidence of selfie-related deaths or injuries. It may be necessary for land-managing organizations, such as national parks and local councils, to devise specific communication and engineering strategies that are tailored to their locales to prevent selfie-related injuries and deaths. There is also a need for researchers to engage with land managers to better evaluate the problem and acquire specific data on selfie incidents rather than relying on media reports.

### Strengths and Limitations

This study is the first to consider how selfie-related deaths at aquatic locations in Australia and the United States are reported and whether there is a focus or not on prevention. We used multiple data sources to gain a clear understanding of the selfie problem as it currently stands through a public health and injury prevention lens. This study did not have access to coronial data from Australia or the United States. The authors intend to access coronial data and incident data (from lifesaving services and national parks) to further assess the selfie problem with an epidemiological study. The authors are not aware of comprehensive data that detail all selfie-related deaths and injuries in Australia or the United States, and due to the media interest in selfie incidents, this was seen as a useful avenue to investigate the problem. However, the study only considered media reports published in English and not behind a paywall and only considered cases in Australia and the United States.

There is an urgent need for better data to be recorded and acquired on selfie injuries and deaths potentially in the form of a prospective registry. Not all deaths or injuries related to selfies are reported or tracked in a consistent manner. It could be the case that many more selfie-related incidents occur but are not reported. There is also a need to engage with land managers when devising preventive approaches to address the issue.

It is important to note that this study specifically focused on selfie taking, which is just one type of mobile phone–based distraction. Future research should consider other types of social media interactions that can lead to potentially hazardous behavior and outcomes.

### Conclusions

Selfie-related incidents are a public health problem that is not merely a social fad or trend. It is imperative that practitioners work with land management agencies to address the issue to avert further injuries and deaths, particularly in aquatic locations. This study found that falls from cliff edges were the most common cause of selfie-related deaths in Australia and the United States in aquatic locations, and that young women and girls were disproportionately affected. This suggests that messaging and prevention efforts should target this demographic and focus on the dangers of taking selfies in natural, scenic areas. The study also found that the media played an important role in reporting on selfie-related incidents but did not convey appropriate education or prevention messages. The study highlights the need for effective prevention strategies to address the problem of selfie-related deaths and injuries in aquatic locations. This could include campaigns to raise awareness about the risks associated with taking selfies in these locations as well as the installation of barriers and warning signs.

## Appendix

Multimedia Appendix 1 Coding framework for content analysis of news reports.

Multimedia Appendix 2 PRISMA (Preferred Reporting Items for Systematic Reviews and Meta-Analyses) checklist.

Multimedia Appendix 3 News media articles reviewed.

## Data Availability

All data used in this study are publicly available. The news reports analyzed for this research have been provided in the Multimedia Appendix 3. The academic research papers that were reviewed have been cited.
