# Supplementary material for: Selfie-Related Incidents: Narrative Review and Media Content Analysis
Source: J Med Internet Res. 2023 Sep 27;25:e47202. doi: 10.2196/47202 (PMC10568398; doi:10.2196/47202)
Supplement: Multimedia Appendix 1 [file jmir_v25i1e47202_app1.docx]

**Multimedia Appendix 1.** Coding framework for content analysis of news reports.

| Overarching theme/category | Code | Files | References |
| --- | --- | --- | --- |
| Blame | Alcohol | 1 | 3 |
| Blame | Blame tone | 8 | 12 |
| Blame | Defending victim | 3 | 7 |
| Blame | Details on victims activities | 11 | 12 |
| Blame | Details on victims life | 6 | 9 |
| Blame | Drugs | 1 | 2 |
| Blame | Events leading up to selfie | 8 | 11 |
| Blame | Party | 4 | 5 |
| Blame | Reports of online abuse of victims following death or injury | 1 | 1 |
| Blame | Social media | 4 | 10 |
| Blame | Tourist apathy or ignorance | 4 | 6 |
| Blame/Warning | Risky behaviour | 16 | 27 |
| Blame/warning | Opinion of locals | 3 | 4 |
| Education and Prevention | Education | 1 | 1 |
| Education and Prevention | Emergency services announcements | 7 | 8 |
| Education and Prevention | Prevention activity | 2 | 2 |
| Education and Prevention | Reference to academic article | 2 | 2 |
| Education and Prevention | References to warning signs | 6 | 8 |
| Information | Reference to selfies | 24 | 31 |
| Information | Details on geography of area | 8 | 10 |
| Information | Details on victim's residency | 9 | 10 |
| Information | Friends | 7 | 13 |
| Information | News inaccuracies | 1 | 3 |
| Information | Time of day | 5 | 6 |
| Information | Victim's family activism | 1 | 2 |
| Information | Where victim was found | 7 | 8 |
| Warning | Reference to other selfie incidents | 9 | 17 |
| Warning | Warning tone | 7 | 12 |
