# Supplementary material for: Selfie-Related Incidents: Narrative Review and Media Content Analysis
Source: J Med Internet Res. 2023 Sep 27;25:e47202. doi: 10.2196/47202 (PMC10568398; doi:10.2196/47202)
Supplement: Multimedia Appendix 3 [file jmir_v25i1e47202_app3.docx]

**Multimedia Appendix 3. News media articles reviewed.**

| Report Number | Media article source | Date accessed | Case ID number | Date published | News site | News site country | Details on signs/barriers and prevention activities in media reports |
| --- | --- | --- | --- | --- | --- | --- | --- |
| 1 | <https://nz.news.yahoo.com/sydney-beachgoers-dice-with-death-to-snap-perfect-nature-selfie-30546711.html> | 15 November 2022 | 1 | 12 January 2016 | Yahoo News | New Zealand | None. |
| 2 | <https://nypost.com/2017/03/27/teen-cheerleader-dies-taking-selfies-in-freak-beach-accident/> | 15 November 2022 | 2 | 27 March 2017 | New York Post | USA | None. |
| 3 | <https://nypost.com/2018/05/18/friends-watch-as-man-falls-to-his-death-taking-selfie-at-tourist-hot-spot/> | 15 November 2022 | 3 | 18 May 2018 | New York Post | USA | Mentions staying on the viewing platform. |
| 4 | <https://www.abc.net.au/news/2018-05-18/instagram-warning-to-tourists-in-quest-for-perfect-holiday-snap/9776060> | 15 November 2022 | 3 | 18 May 2018 | ABC News | Australia | References viewing platform. |
| 5 | <https://www.abc.net.au/news/2018-07-24/kurnell-cliff-fall-mormon-missionary-gavin-zimmerman/10029406> | 15 November 2022 | 4 | 24 Jul 2018 | ABC news | Australia | References a police warning to stay on the viewing platform. |
| 6 | <https://www.abc.net.au/news/2018-07-23/whale-watching-tourist-dies-after-falling-off-cliff/10026016> | 15 November 2022 | 4 | 23 Jul 2018 | ABC News | Australia | Questions whether there were any preventive measures in place. |
| 7 | https://www.stuff.co.nz/world/australia/105704065/man-falls-20m-to-death-taking-selfies-at-cliffs-edge | 15 November 2022 | 4 | 24 July 2018 | Stuff | New Zealand | Warns people to stay on viewing platform. |
| 8 | <https://www.boreal.org/2018/09/21/180544/woman-who-died-at-pictured-rocks-was-taking-selfie-fell-off-cliff> | 15 November 2022 | 5 | 21 September 2018 | Boreal | USA | None. |
| 9 | <https://www.freep.com/story/news/nation-now/2018/09/21/pictured-rocks-death-tu-thanh-nguyen-lake-superior/1382807002/> | 15 November 2022 | 5 | 21 September 2018 | Detroit Free Press | USA | None. |
| 10 | <https://abc11.com/man-falls-into-potomac-river-while-trying-to-take-selfie/4393585/> | 15 November 2022 | 6 | 3 October 2018 | ABC11 | USA | None. |
| 11 | https://www.cbsnews.com/baltimore/news/man-taking-selfie-falls-into-rushing-waters-of-potomac-river/ | 15 November 2022 | 6 | 1 OCTOBER 2018 | CBS News | USA | None. |
| 12 | <https://www.abc10.com/article/news/woman-dies-taking-selfie-at-south-lake-tahoes-eagle-falls/103-b4825fd3-9f91-41a7-be2e-caf9e74e8963> | 15 November 2022 | 7 | 31 May 2019 | ABC 10 News | USA | None. |
| 13 | https://www.mercurynews.com/2019/06/01/woman-taking-selfie-photo-dies-after-falling-over-edge-of-eagle-falls-in-emerald-bay/ | 15 November 2022 | 7 | 1 June 2019 | Mercury News | USA | None. |
| 14 | https://www.sfgate.com/renotahoe/article/woman-dies-selfie-Lake-Tahoe-Eagle-Falls-Emerald-13915770.php | 15 November 2022 | 7 | 1June 2019 | San Francisco Gate | USA | None. |
| 15 | <https://7news.com.au/news/disaster-and-emergency/woman-dies-after-falling-from-sydney-cliff-c-404355> | 15 November 2022 | 8 | 17 August 2019 | 7News | Australia | References the installation of CCTV, more physical barriers, multilingual signage and more frequent ranger patrols. |
| 16 | <https://www.smh.com.au/national/nsw/woman-dies-at-selfie-spot-in-sydney-s-east-20190817-p52i3p.html> | 15 November 2022 | 8 | 17 August 2019 | Sydney Morning Herald | Australia | Recommends personal responsibility, obeying signage and sticking to paths. |
| 17 | https://www.theguardian.com/australia-news/2019/aug/17/sydney-cliff-fatality-woman-falls-to-death-at-popular-selfie-spot | 15 November 2022 | 8 | 17 August 2019 | The Guardian | Australia | Mentions restricting or deterring movement, installing CCTV, providing barriers and multilingual signage, and ranger patrols. |
| 18 | <https://www.bbc.com/news/uk-england-lincolnshire-51103837> | 15 November 2022 | 9 | 14 January 2020 | BBC News | UK | Mentions signage. |
| 19 | <https://euroweeklynews.com/2020/01/13/british-model-21-falls-to-her-death-from-cliff-top-selfie-spot-in-sydney/> | 15 November 2022 | 9 | 13 January 2020 | Euro Weekly News | Spain | None. |
| 20 | <https://people.com/human-interest/madalyn-davis-dies-australia-selfie-cliff/> | 15 November 2022 | 9 | 14 January 2020 | People | USA | References signs. |
| 21 | <https://www.dailymail.co.uk/news/article-8227841/Teenage-girl-injured-falling-selfie-cliff-Diamond-Bay-tourist-died-January.html> | 15 November 2022 | 10 | 17 April 2020 | Daily Mail | Australia | References a fence. |
| 22 | <https://www.bbc.com/news/uk-england-lincolnshire-55401817> | 15 November 2022 | 10 | 21 December 2020 | BBC News | UK | None. |
| 23 | https://www.dailymail.co.uk/news/article-9763527/Kangaroo-Point-tragedy-Brazilian-woman-33-dies-accidentally-falling-cliff.html | 15 November 2022 | 11 | 7 July 2021 | Daily Mail | UK | None. |
| 24 | <https://www.independent.co.uk/news/world/americas/georgia-hiker-selfie-waterfall-rescue-b2085710.html> | 15 November 2022 | 12 | 23 May 2022 | The Independent | UK | None. |
